# Ageing Increases Vulnerability to Aβ42 Toxicity in *Drosophila*

**DOI:** 10.1371/journal.pone.0040569

**Published:** 2012-07-12

**Authors:** Iain Rogers, Fiona Kerr, Pedro Martinez, John Hardy, Simon Lovestone, Linda Partridge

**Affiliations:** 1 Institute of Healthy Ageing and GEE, University College London, London, United Kingdom; 2 Institute of Neurology, University College London, London, United Kingdom; 3 MRC Centre for Neurodegeneration Research, Institute of Psychiatry, King's College London, London, United Kingdom; 4 Max Planck Institute for Biology of Ageing, Köln, Germany; Thomas Jefferson University, United States of America

## Abstract

Age is the major risk factor for many neurodegenerative diseases, including Alzheimer's Disease (AD), for reasons that are not clear. The association could indicate that the duration or degree of exposure to toxic proteins is important for pathology, or that age itself increases susceptibility to protein toxicity. Using an inducible *Drosophila* model of AD, we investigated these possibilities by varying the expression of an Aβ42 transgene in neurons at different adult ages and measuring the effects on Aβ42 levels and associated pathological phenotypes. Acute induction of Arctic Aβ42 in young adult flies resulted in rapid expression and clearance of mRNA and soluble Arctic Aβ42 protein, but in irreversible expression of insoluble Arctic Aβ42 peptide. Arctic Aβ42 peptide levels accumulated with longer durations of induction, and this led to a dose-dependent reduction in negative geotaxis and lifespan. For a standardised level of mRNA expression, older flies had higher levels of Arctic Aβ42 peptide and associated toxicity, and this correlated with an age-dependent reduction in proteasome activity. Equalising Aβ42 protein at different ages shortened lifespan in correlation with the duration of exposure to the peptide, suggesting that Aβ42 expression accumulates damage over time. However, the relative reduction in lifespan compared to controls was greater in flies first exposed to the peptide at older ages, suggesting that ageing itself also increases susceptibility to Aβ42 toxicity. Indeed older flies were more vulnerable to chronic Aβ42 toxicity even with a much lower lifetime exposure to the peptide. Finally, the persistence of insoluble Aβ42 in both young and old induced flies suggests that aggregated forms of the peptide cause toxicity in later life. Our results suggest that reduced protein turnover, increased duration of exposure and increased vulnerability to protein toxicity at later ages in combination could explain the late age-of-onset of neurodegenerative phenotypes.

## Introduction

Ageing is the major risk factor for common, chronic, killer conditions, including cancer, cardiovascular disease and neurodegeneration. The aetiology of many neurodegenerative diseases includes the formation of toxic protein aggregates in neurons. For instance Alzheimer's disease (AD), the most prevalent form of senile dementia, is characterised by the widespread presence of extracellular amyloid plaques, predominantly composed of amyloid beta (Aβ) peptides, and intraneuronal neurofibrillary tangles formed from insoluble fibrillar aggregates of the microtubule-binding protein tau [1]. Most cases of AD (>99%) are sporadic [2], with age as the main risk factor [3], [4].

A large body of evidence suggests that the accumulation and deposition of Aβ peptides are the primary influence driving the disease [5], [6]. Support for this ‘amyloid cascade’ hypothesis comes from mutations causing early-onset, familial AD (FAD), which affect the amyloid precursor protein (APP), from which Aβ peptides are derived, and presenilins PS1 and PS2, which are involved in the cleavage of APP to yield Aβ peptides. These mutations increase production of Aβ, levels of Aβ42 relative to Aβ40 or the propensity of Aβ42 to aggregate (for review see [7]). In addition, many mouse models of AD, typically based on the overexpression of FAD associated APP alone or in combination with mutations in PS1, develop age-dependent Aβ plaques and behavioral and memory deficits [8]. Furthermore, studies in the fruit fly *Drosophila melanogaster* [9], [10], [11], [12] and the nematode worm *Caenorhabditis elegans* [13], [14] have demonstrated that direct expression of the toxic Aβ42 peptide leads to an age-dependent accumulation of Aβ42, neuronal dysfunction and shortened lifespan.

Other neurodegenerative conditions share both the aggregation of toxic protein and a late age of onset [15]. For instance, Parkinson's Disease is associated with aggregation of α-synuclein in Lewy bodies, with most cases sporadic and age the main risk factor [16]. However the mechanisms linking protein aggregation and the appearance of disease to age remain to be identified. Protein aggregation may accumulate to a toxic threshold, or the duration and extent of exposure may be important for inducing neuronal dysfunction. In addition, the ageing process itself could increase vulnerability to toxic proteins by impairing their clearance or increasing vulnerability to their toxic effects [15], [17]. All of these factors could be important – they are not mutually exclusive.

To investigate the mechanisms linking protein toxicity to age, we have induced expression of a toxic protein at different ages in neurons of adult *Drosophila*. If ageing plays a direct role then we would expect that standardised exposure of older neurons to a transgene encoding a toxic protein would lead either to greater levels of toxic protein, or greater toxicity of a standard dose of protein, in older flies. The few studies that have addressed this issue have suggested that ageing is indeed important. Brewer et al., (1998) isolated neurons from embryonic, young and old-aged rat hippocampus and exposed them to toxic Aβ fragments (25–35) finding that toxicity, as measured by cell death, was age-, dose- and time-dependent. Guela et al., (1998) demonstrated that aged rhesus monkeys were more vulnerable than young to injected plaque-equivalent concentrations of fibrillar Aβ, with the young monkeys developing no pathology at all. However, these studies used either extracellular application of Aβ fragments or microinjection of fibrillar Aβ to induce toxicity, which makes some assumptions about the nature and site of Aβ toxicity. Evidence is increasing that intracellular [18], soluble oligomers of Aβ, rather than extracellular, fibrillar forms of the peptide, are pathogenic [19]. Furthermore, ageing could increase vulnerability to Aβ toxicity through the mechanisms by which the peptide is produced and broken down. Hence, we have taken advantage of powerful systems for conditional and tissue-specific gene over-expression in *Drosophila* [20] to examine the effects of age on Aβ peptide toxicity under physiological conditions *in vivo*.

We have previously shown that induced expression of the FAD-associated Arctic Aβ42 (Arc Aβ42) isoform in adult neurons of *Drosophila* results in age-dependent locomotor and electrophysiological deficits and shortened lifespan [21]. In the present study, we show that these pathological phenotypes are dependent on the concentration of Aβ42. For equivalent Aβ42 mRNA levels, higher levels of Arc Aβ42 peptide were present in older flies, and this correlated with an age-dependent reduction in proteasome activity and resulted in shortened lifespan. Controlling for this age-dependent reduction in protein turnover, expression of equivalent amounts of Aβ42 protein at different ages shortened lifespan in correlation with the duration of exposure to the peptide. This suggests that the relationship between Aβ42 toxicity and age reflects an accumulation of damage over time. Despite this, however, the relative reduction in lifespan compared to non-Arctic Aβ42-expressing controls was greater in flies first exposed to the peptide at older ages, suggesting that ageing itself also increases susceptibility to Aβ42 toxicity. Indeed older flies were more vulnerable to death from even much lower levels of exposure to Aβ42 protein. Our results therefore suggest that increasing age is associated with greater protein toxicity through a combination of reduced clearance, increased exposure and increased vulnerability to pathogenic proteins.

## Results

### Dynamics of Aβ42 expression and toxicity using the elavGS-UAS system

**Figure 1:**
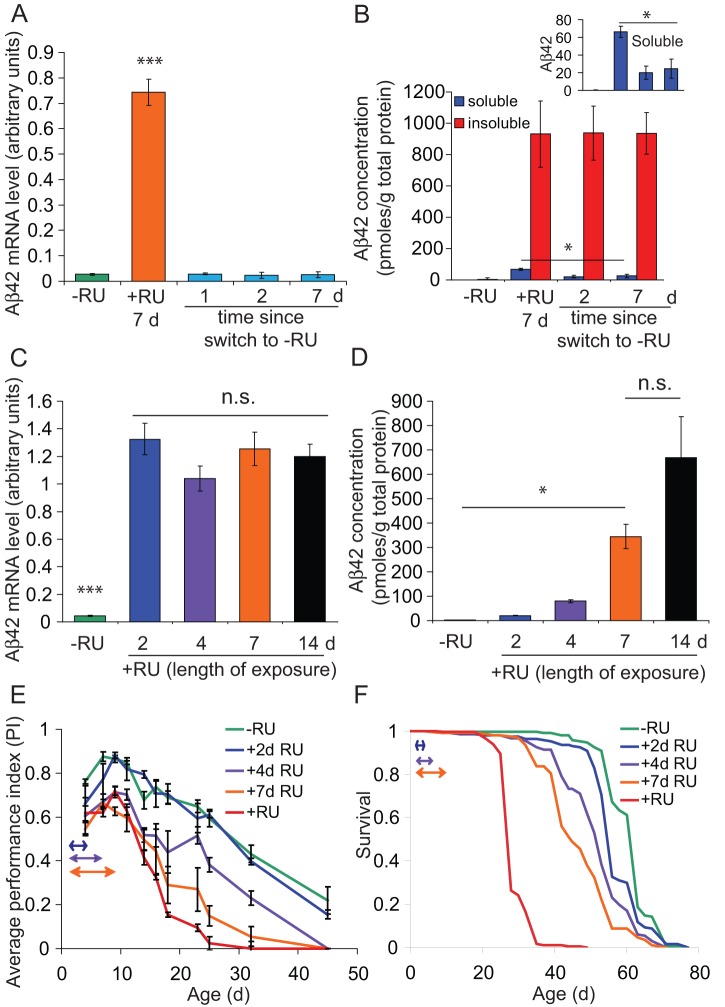
Effects of varying RU486 exposure time on Aβ42 levels and toxicity. 2-day-old UAS-Arc Aβ42/+; elavGS/+ females were treated with 200 µM RU486 for 2, 4, 7 or 14 days before being transferred to RU486-free medium (see table S1 for pulse conditions). Flies maintained chronically on – RU486 (−RU) and 200 µM RU486 (+RU) were used as negative and positive controls, respectively. Data are presented as means ± SEM and were analysed by ANOVA followed by Tukey's honestly significant difference (HSD) post-hoc test. (A & B) Removal of RU486 resulted in rapid clearance of Arctic Aβ42 mRNA as well as soluble, but not insoluble, protein levels. (A) Aβ42 mRNA levels were measured by quantitative RT PCR prior to RU486 treatment (−RU), at the end of treatment (+RU 7 d), and at the indicated time-points following the switch to RU486-free food (see methods). ***P<0.001 comparing the level of Arctic Aβ42 mRNA at the end of RU treatment to all other conditions (n = 3). (B) Soluble and insoluble Arctic Aβ42 was fractionated (see methods) and measured by ELISA at the indicated time-points (n = 3). *P*<0.0001 comparing soluble and insoluble Aβ42 fractions (two-way ANOVA). Soluble Aβ42 was reduced to baseline levels following switch-off (see inset; *P<0.05 comparing +RU 7d to 2 or 7d following transfer to −RU food and no significant difference comparing 2 or 7d following switch to −RU to non-RU-treated controls; Tukey's HSD), whereas insoluble Aβ42 was highly stable for 1 week following cessation of transgene expression (no significant difference between +RU 7d and 2 or 7d following switch to −RU, Tukey's HSD). (C–F) Arctic Aβ42 protein levels correlate with increasing duration of RU486 exposure and associate with impairments in function. (C) Arctic Aβ42 mRNA levels were quantified at the end of each RU486 pulse, as indicated. Aβ42 transcript was significantly increased under all RU treatment conditions compared to untreated controls (***P<0.001, n = 5, Tukey's HSD), with no significant difference (n.s.) between the varying RU486 pulse conditions. (D) Total (soluble and insoluble) Aβ42 peptide was extracted using Guanidine HCl and levels were measured by ELISA at the end of each RU486 pulse (see methods). Total Arctic Aβ42 protein levels increased with the length of RU486 exposure (*P*<0.0001, one-way ANOVA, n = 3). *P<0.05 comparing 2, 4 and 7 day pulses, but no significant difference between 7 and 14 day pulses. *P*<0.05 comparing −RU to all + RU treatments conditions (Tukey's HSD). (E) Climbing ability was assessed at the indicated time-points. Arrows indicate the period of RU486 treatment for each pulse. Data were analysed by two-way ANOVA and Tukey's HSD (n = 3, number of flies per group = 39–45). Negative geotaxis decreased with increasing RU486 exposure length. Pulse lengths ≥4 days were significantly different from −RU controls, whereas pulse lengths ≤4 days were significantly different from chronically treated (+RU) controls (*P*<0.05, Tukey's HSD). A 2-day pulse of RU486 did not significantly induce climbing defects compared to untreated flies, whereas a 7-day pulse did not significantly improve the reduced climbing ability compared to chronically treated +RU controls. (F) Survival decreased with increasing RU486 exposure time. Median lifespans were: 62 days for –RU (chronic), 55 days for +2d RU, 52 days for +4d RU, 45 days for +7d RU and 27 days for + RU (chronic). All survival curves were significantly different from each other (*P*<0.001, log rank test).

We previously characterised an adult-onset, fly model of Alzheimer's disease [21], generated by expressing the Arctic Aβ42 peptide (UAS-Arc Aβ42; [11]) under the control of an RU486-inducible pan-neuronal driver (elav GeneSwitch (elavGS); [20], [22]). Under chronic induction conditions the levels of Arc Aβ42 mRNA and protein expression appeared to vary with age [21]. Aβ42 RNA expression declined with age, probably reflecting a lower intake of the RU486 inducer as a consequence of the known age-dependent reduction in feeding behaviour [23], whereas protein levels increased with age, reflecting either a time-dependent accumulation of the peptide or an age-dependent reduction in protein turnover, or both. We now show further that, under acute induction conditions, Aβ42 mRNA (Figure 1A) and soluble protein (Figure 1B) levels are rapidly cleared following removal of RU486, whereas aggregated forms of the peptide are stable for up to one week (Figure 1B). Varying the duration of RU486 exposure for 2, 4, 7 or 14 days (see Table S1 for pulse conditions), did not alter the level of Arc Aβ42 mRNA expressed at the end of the induction period (Figure 1C), suggesting that the age-dependent reduction in transcript observed under chronic conditions occurs at ages over 21 days. The concentration of Arc Aβ42 protein increased in correlation with the duration of RU486 exposure (Figure 1D) and this led to progressively more severe reductions in negative geotaxis (Figure 1E) and survival (Figure 1F), presumably due to the persistence of insoluble forms of peptide after cessation of induction of gene expression.

### Age-dependent reduction in protein turnover correlates with Aβ42 peptide accumulation and toxicity

We next aimed to use our inducible model to investigate the intrinsic effects of age on vulnerability to Aβ42 toxicity, by inducing comparable levels of Arc Aβ42 at different ages and measuring the time to develop, and extent of, the subsequent reduction in survival. This output variable allowed us to induce Aβ42 expression at a wide range of ages prior to the onset of ageing-related deaths, unlike negative geotaxis, which starts to decline already in the first week of life. Notably, chronic administration of RU486 at the highest (200 µM) dose used in our study had no effect on lifespan of non-Aβ42-expressing flies (Figure S1), demonstrating that our observed effects of Aβ42 induction on survival are not attributable to the RU486 treatment conditions.

**Figure 2:**
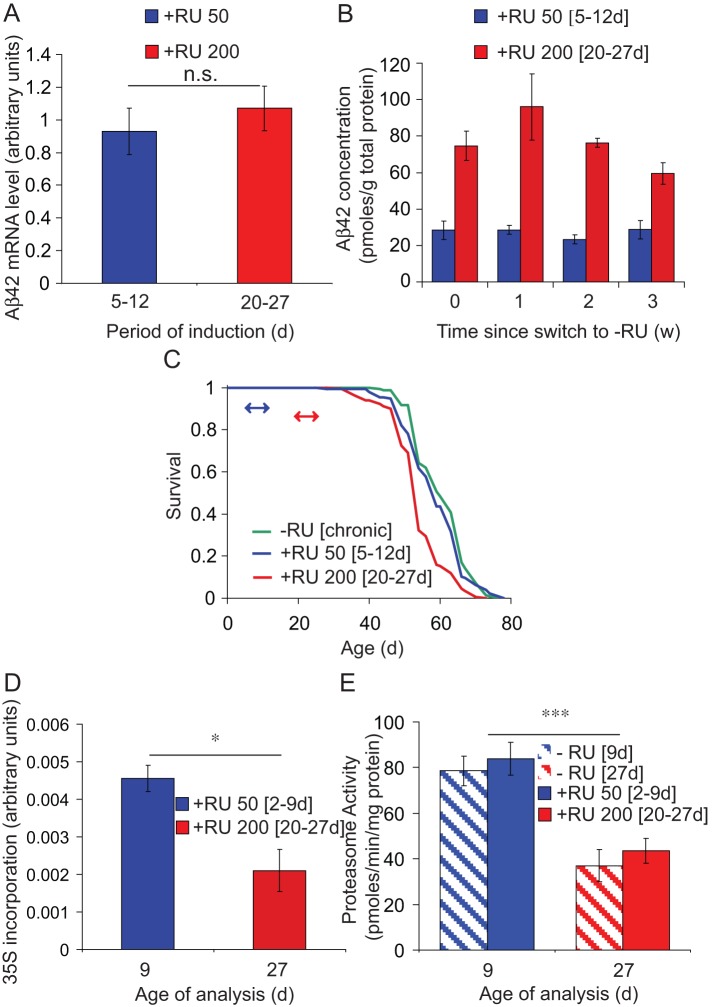
Ageing increases Arctic Aβ42 peptide accumulation and toxicity when transcript levels are equalised at different ages. UAS-Arctic Aβ42/+; elavGS/+ females were pulsed, for 1 week, either at 2 or 5 days old with 50 µM RU (RU 50 [2–9d] or [5–12d]) or 20 days old with 200 µM RU (RU 200 [20–27d]). Aβ42 mRNA, lifespan and protein levels were then measured. Data are presented as means ± SEM. (A) Arctic Aβ42 transcripts were measured at the end of the induction period (see methods). Aβ42 transcript levels were not significantly different between RU 50 [5–12d] and RU 200 [20–27d] flies under these induction conditions (*P* = 0.5, student's t-test, n = 4). A more detailed analysis of Aβ42 expression levels at varying RU486 concentrations can be found in Fig S1. (B) Arctic Aβ42 peptide accumulates in older flies at equivalent levels of Aβ42 mRNA. Total (soluble and insoluble) Aβ42 peptide was extracted using GnHCl (see methods), and levels were measured by ELISA at the end of the RU486 pulse (0d) then 1, 2 and 3 weeks following the switch to RU486-free medium. Data were analysed by two-way ANOVA followed by Tukey's HSD post-hoc (n = 4). RU 200 [20–27d] flies contained significantly more Aβ42 peptide than RU 50 [5–12d] flies at all time-points measured (*P*<0.0001). No significant difference in protein levels was observed up to 3 weeks following switch-off at either age of induction (*P* = 0.382). (C) Equivalent levels of Aβ42 transcripts reduced survival only when induced in old flies (RU 200 [20–27d]). Arrows indicate the period of RU486 treatment. Median lifespans were: 60 days for −RU, 58 days for RU 50 [5–12d], and 53 days for RU 200 [20–27d] flies. *P* = 0.18 comparing −RU to RU 50 [5–12d] flies, *P*<0.0001 comparing −RU to RU 200 [20–27d] flies (log-rank test). (D) Protein translation, as measured by ^35^S-methionine incorporation, decreases with age in heads of flies conditionally over-expressing Arctic Aβ42. Data were analysed by Student's t-test (*P<0.05, comparing RU 50 [2–9d] to RU 200 [20–27d] flies, n = 5). (E) Proteasome activity, as measured using the fluorogenic peptide substrate LLVY-AMC, is reduced with age in the heads of flies conditionally over-expressing Arctic Aβ42 as well as non-RU-treated controls. Data are presented as mean activities (pmoles/min/mg protein) ± SEM (n = 5). *** *P*<0.0001 comparing 9 versus 27 day-old flies, *P* = 0.429 comparing + versus – RU486 (two-way ANOVA); *P*<0.05 comparing RU 50 [2–9d] to RU 200 [20–27d] flies and –RU day 9 to –RU day 27 flies (Tukey's HSD).

Our analysis of the dynamics of Aβ42 expression, as described above, suggested that to standardise Aβ42 levels at different ages both the concentration of RU486 and the length of exposure to the inducer must be finely tuned. We first equalised expression levels of Aβ42 mRNA in flies at day 5 or day 20 post-eclosion, by varying the concentration of RU486, inducing for 1 week and measuring transcript levels at the end of the induction period (Figure S2). Induction at all RU486 concentrations produced significant (*P*<0.05) Aβ42 over-expression, and levels of Aβ42 mRNA were similar in flies induced at day 5 with 50 µM (RU 50 [5–12d]) and at day 20 with 200 µM (RU 200 [20–27d]) RU486, respectively (Figures 2A and S2). However, these standardised levels of mRNA expression led to different levels of Aβ42 protein, measured at the end of the 1-week pulse (0d) and 1, 2 and 3 weeks following switch-off (Figure 2B). Total Arctic Aβ42 peptide levels were significantly higher (*P*<0.0001, two-way ANOVA) in RU 200 [20–27d] than in RU 50 [5–12d] induced flies, and no significant reduction occurred for up to 3 weeks following switch-off in either age group, further confirming that Arctic Aβ42 peptide is highly resistant to degradation. Presumably associated with the higher levels of Arctic Aβ42 peptide present in the day-20-induced flies (RU 200 [20–27d]), only they displayed a shortened lifespan in response to Aβ42 expression (Figure 2C).

For a given level of mRNA, older flies thus had higher levels of Aβ42 peptide. Protein turnover declines with age in several organisms 24,25, suggesting that age-dependent effects on protein synthesis or degradation could explain the accumulation of Aβ42 peptide in older flies. As a measure of the rate of translation, ^35^S-methionine incorporation was assessed in day-2 (RU 50 [2–9d]) and day-20 (RU 200 [20–27d]) UAS-Arc Aβ42; elavGS flies pulsed with RU486 for 1 week. ^35^S-methionine incorporation was significantly lower in RU 200 [20–27d] flies, indicating a reduced rate of translation (Figure 2D). An increase in protein synthesis is, therefore, unlikely to explain the accumulation of Arc Aβ42 peptide in older flies. However, proteasome activity was also significantly reduced in RU 200 [20–27d] compared to RU 50 [2–9d] Arc Aβ42 over-expressing flies, as well as in older non-RU-treated flies (Figure 2E) and elavGS driver flies alone under the same treatment conditions (Figure S3), suggesting a general age-dependent reduction in proteasomal degradation which may account for the higher levels of the Aβ42 peptide following induction in older flies and, at least in part, for the consequently increased toxicity.

### Duration of exposure and ageing *per se* increase vulnerability to insoluble Arctic Aβ42 peptide

To measure the vulnerability of older flies to protein toxicity *per se*, we next aimed to control for changes in the rate of protein turnover with age by producing standardised levels of Aβ42 peptide at different ages. UAS-Arc Aβ42; elavGS flies were treated with a range of RU486 doses (50–200 µM RU) for varying exposure times (1 or 2 weeks) from 5, 20 and 30 days of age. At the end of the induction period, total Aβ42 peptide concentration was measured (Figure S4). A variety of treatments resulted in equivalent levels of Aβ42 peptide at different ages (Figure S4; non-significant difference in Aβ42 peptide levels); (1) a 2 week pulse of 100 µM RU in 5-day old flies (RU 100 [5–19d]) versus 200 µM RU in 20-day (RU 200 [20–34d]) and 30-day (RU 200 [30–44d]) old flies and (2) a 1 week pulse of 200 µM RU in 5-day (RU 200 [5–12d]) versus 20-day (RU 200 [20–27d]) old flies.

**Figure 3:**
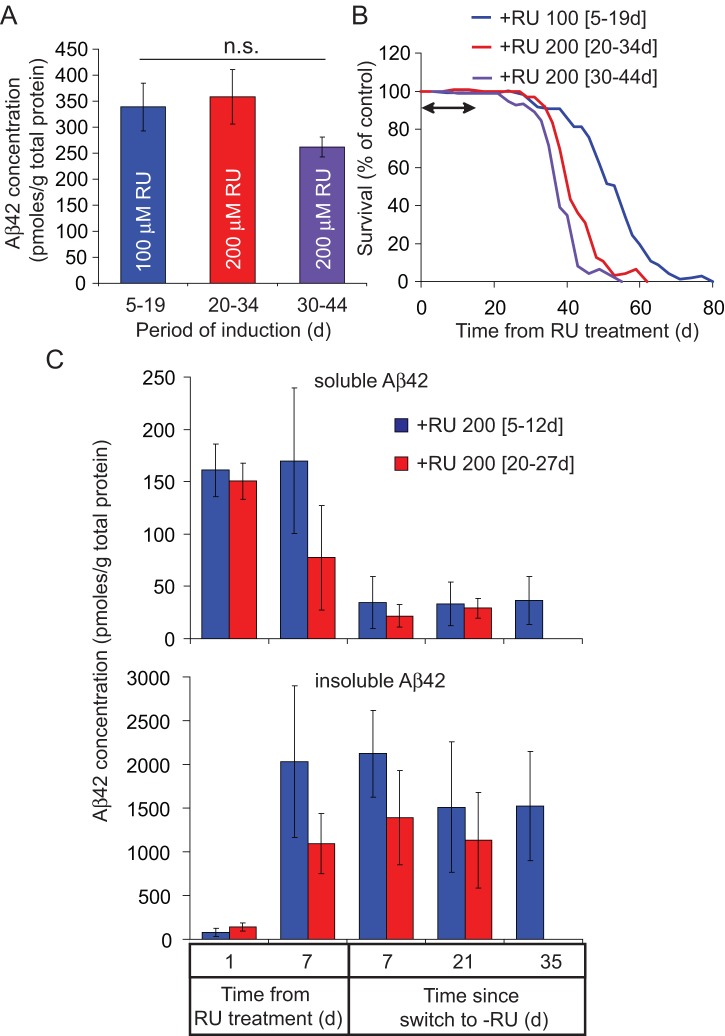
Older flies are more vulnerable to a standardised dose of Arctic Aβ42 peptide. (A & B) UAS-Arctic Aβ42/+; elavGS/+ females were treated, for 2 weeks, with 100 µM RU486 from 5 days (RU 100 [5–19d]) and 200 µM RU486 from 20 (RU 200 [20–34d]) and 30 (RU 200 [30–44d]) days post-eclosion, and were maintained on RU486-free medium prior to and following the pulse period. (A) Total Aβ42 protein levels (soluble and insoluble as measured by GnHCl extraction) were equivalent at the end of the RU pulse when comparing each age of induction. Data are expressed as means ± SEM (n = 5) and were compared using one-way ANOVA and Tukey's HSD post-hoc. No significant differences were observed. (B) Survival, expressed as a percentage of non-RU486-treated controls, plotted from the age of RU486 induction. Arrow indicates the induction period. Older flies exhibit a significantly greater reduction in relative survival following RU treatment (*P*<0.05 comparing RU 200 [20–34d] and RU 200 [30–44d] with RU 100 [5–19d] and RU 200 [20–34d] with RU 200 [30–44d], Wilcoxon matched pairs sign rank test). Raw survival curves are depicted in Fig S4A. (C) UAS-Arctic Aβ42/+; elavGS/+ females were pulsed for 1 week with 200 µM RU486 from 5 (RU 200 [5–12d]) and 20 (RU 200 [20–27d]) days post-eclosion, to equalise the total Aβ42 level in young versus old-induced flies (see Fig S3). Soluble and insoluble Aβ42 was fractionated (see methods) and measured by ELISA at 1 and 7 days following induction, then 7, 21 and, in the case of RU 200 [5–12d] flies, 35 days following the switch to RU486-free food. Data are expressed as means ± SEM and were analysed by two-way ANOVA (n = 4). No significant differences were observed comparing RU 200 [5–12d] versus RU 200 [20–27d]-induced flies for either soluble (*P* = 0.428) or insoluble (*P* = 0.258) Aβ42 fractions. Equivalent levels of soluble and insoluble Aβ42 were observed 1 day following induction (n.s. difference between insoluble Aβ42 at day 1 versus soluble Aβ42 at days 1 and 7 following RU treatment). Soluble Aβ42 levels did not vary significantly during the induction period (*P* = 0.546 comparing flies on RU486 for 1 day versus 7 days). Soluble Aβ42 was significantly reduced following switch-off (*P*<0.001 comparing flies at the end of the 7-day induction period with flies switched to −RU food), reaching baseline levels within 7 days (*P* = 0.99 comparing RU 200 [5–12d] flies switched to −RU food for 7, 21 and 35 days and *P* = 0.628 comparing RU 200 [20–27d] flies switched to −RU for 7 and 21 days). Insoluble Aβ42 levels increased significantly during the RU486 induction period (*P* = 0.016 comparing flies on RU486 for 1 day versus 7 days, *P*<0.05 comparing insoluble Aβ42 levels in flies on RU for 7 days versus soluble Aβ42 levels at days 1 and 7 following induction). Insoluble Aβ42 was not significantly reduced following switch-off (*P* = 0.886 comparing flies at the end of the 7-day induction period with flies switched to −RU food) and remained stable for several weeks following transfer to RU486-free food (*P* = 0.744 comparing RU 200 [5–12d] flies switched to −RU food for 7, 21 and 35 days and *P* = 0.748 comparing RU 200 [20–27d] flies switched to −RU for 7 and 21 days).

Arctic Aβ42 toxicity was assessed by measuring survival under the RU486 treatment conditions described in (1) above (Figures 3 and S5A). Equivalent levels of Aβ42 peptide expression were again confirmed at the end of the 2 week induction period in day-5, day-20 and day-30-induced flies (Figur 3A). All RU-treated flies had reduced median lifespan compared to non-RU-treated controls: 27% for RU 100 [5–19d], 24% for RU 200 [20–34d] and 14% for RU 200 [30–44d] (Figure S5A). Measuring survival from 44 days only, when Aβ42 peptide (which once present is not cleared) and age are equivalent in all treatment groups, young-induced flies had a shorter life expectancy compared to old-induced flies (Figure S5B). This suggests that susceptibility to Aβ42 toxicity correlates with the duration of exposure to the peptide, most probably reflecting an accumulation of damage over time. To isolate the effect of age specifically on vulnerability to Arc Aβ42 toxicity, further data analysis controlled for both the duration of exposure to insoluble Arc Aβ42 protein and the direct effect of age on survival (Figure 3B), with older flies expected to die sooner even in the absence of any Arc Aβ42. To control for the longer exposure to Aβ42 in younger flies, we re-analysed survival as function of time from the age of induction. To control for the direct effect of age on survival, we expressed survival as a percentage of that of non-RU-treated controls at each time-point from the age of induction. Relative survival following RU486 treatment was progressively lower in the two groups of older flies (Figure 3B; *P*<0.05 comparing RU 100 [5–19d] flies to RU 200 [20–34d] and RU 200 [30–44d] flies; *P*<0.05 comparing RU 200 [20–34d] and RU 200 [30–44d] flies, Wilcoxon rank sign test), suggesting that ageing itself also increases vulnerability to toxicity from a standard dose of Aβ42 peptide. Finally, measuring relative survival of RU 100 [5–19d] flies from progressively later time-points (Figure S5C) revealed that flies over-expressing Arctic Aβ42 early in life were more severely disadvantaged in their survival compared to controls at older ages. This reduced survival with age may be attributable to an accumulation of damage with time, as indicated by the increase in toxicity with longer exposures to Aβ42 peptide (Figure S5B), or an ageing-dependent increase in susceptibility to Aβ42 toxicity (Figure 3B), or both.

We next examined whether the increased vulnerability of older flies to Aβ42 toxicity was due to differences in protein aggregation with age. To standardise the dose of total Aβ42 peptide at different ages, UAS-Arc Aβ42; elavGS flies were induced for 1 week as described in (2) above. Soluble and insoluble proteins were then separated and Aβ42 measured in each fraction at 1 and 7 days after induction, then at 7, 21 and, in the case of RU 200 [5–12d]-induced flies, 35 days following switch-off (Figure 3C). Equivalent levels of Aβ42 were detected in soluble and insoluble pools immediately following RU treatment (no significant difference comparing insoluble Aβ42 at 1 day versus soluble Aβ42 at 1 and 7 days following RU486 treatment). Aβ42 aggregated during the induction period with a significant increase in insoluble Aβ42 between 1 and 7 days following RU treatment (*P* = 0.016 comparing flies on RU for 1 day versus 7 days). Soluble Aβ42 was cleared to baseline levels within 1 week following switch-off, but insoluble protein remained stable with no significant reduction in peptide levels for several weeks following transfer to RU486-free food (*P* = 0.886 comparing flies switched to –RU486 food to those at the end of the 7 day induction period). No significant difference in the rate of Aβ42 aggregation or clearance was observed between young and old-induced flies (*P* = 0.428 comparing soluble, and *P* = 0.258 comparing insoluble, Aβ42 levels between RU 200 [5–12d] and RU 200 [20–27d]-induced flies). Hence, older flies were more susceptible to the same form of Aβ42 as is present in young flies. Moreover, the majority of Arc Aβ42 was insoluble at late time-points in both RU 200 [5–12d] and RU 200 [20–27d]-induced flies, suggesting that insoluble forms of the peptide are involved in inducing toxicity later in life.

### Older flies are more vulnerable to chronic Aβ42, despite a lower lifetime exposure to the peptide

**Figure 4:**
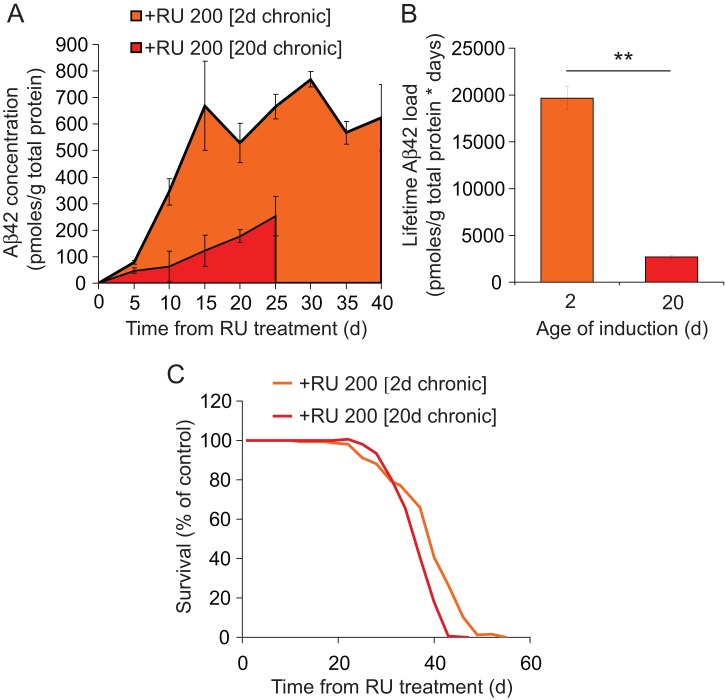
Older flies are more vulnerable to chronically induced Arctic Aβ42, despite a lower lifetime exposure to the peptide. (A) UAS-Arctic Aβ42/+; elavGS/+ females were chronically treated with 200 µM RU486 from 2-days (RU 200 [2d chronic]) and 20-days (RU 200 [20d chronic]) post eclosion. Total (soluble and insoluble) Arctic Aβ42 protein levels were assayed every 5 days until flies started dying. Data are presented as means ± SEM and were analysed by two-way ANOVA and Tukey's HSD (n = 3). Aβ42 levels were higher in RU 200 [2d chronic] versus RU 200 [20d chronic] flies at all time-points up to 25 days post-induction (*P*<0.05, Tukey's HSD) except day 5. (B) A crude measure of the lifetime Arctic Aβ42 load (from induction until the start of decline in survival) was determined in 2d and 20d-induced flies by calculating the area under the curves in (A). Data are presented as the means ± SEM (n = 3). The lifetime Arctic Aβ42 load was significantly lower in RU 200 [20d chronic] than in RU 200 [2d chronic]-treated flies (**P<0.01, student's t-test). (C) Survival, expressed as a percentage of non-RU486-treated controls, plotted from the age of RU486 induction. RU 200 [20d chronic] flies exhibited a significant reduction in relative survival following RU486 treatment compared to RU 200 [2d chronic] flies (*P*<0.05, Wilcoxon matched pairs sign rank test). Original survival curves are depicted in Fig S4B.

Finally, we examined the effects of age on Aβ42 toxicity when induced chronically, to allow protein to accumulate continuously under conditions that may more closely re-capitulate the disease situation. UAS-Arctic Aβ42; elavGS flies were induced from 2 days and 20 days post-eclosion and total Arctic Aβ42 protein levels assayed every 5 days until death (Figure 4A & B). Arctic Aβ42 accumulated following induction in both RU 200 [2d chronic] and RU 200 [20d chronic]-induced flies, but at a slower rate and to a much lower maximum concentration in older flies (Figure 4A). This most probably reflects reduced induction of gene expression caused by age-dependent reductions in feeding behaviour, and therefore consumption of inducer, in older flies. The area under the curve of Aβ42 accumulation across time was calculated as a crude measure of lifetime exposure to Aβ42 in RU 200 [2d chronic] and RU 200 [20d chronic]- induced flies, revealing that old-induced flies were exposed to 7.3 fold lower Aβ42 from induction until death than young-induced flies (Figure 4B).

Survival was significantly more reduced in RU 200 [2d chronic] than in RU 200 [20d chronic]-treated flies (Figure S5B, 45% versus 25%, *P*<0.05, log rank test), possibly due to either the longer duration of exposure or increased concentration of the peptide in young-induced flies. However, as described above, we controlled for the longer exposure to Aβ42 in young-induced flies, by re-calculating survival as function of the time from the age of induction, and for the effects of age on survival by expressing survival as a percentage of non-RU-treated controls at each time-point (Figure 4C). Remarkably, RU 200 [20d chronic]-induced flies exhibited a significantly greater reduction in relative survival than did RU 200 [2d chronic]-induced flies (*P*<0.05, Wilcoxon matched pairs sign rank test). Since old-induced flies were exposed to much lower maximum and cumulative amounts of Aβ42 peptide, these results further support the conclusion that older flies are more vulnerable to Arctic Aβ42 protein toxicity.

## Discussion

Many neurodegenerative diseases share in common the accumulation of toxic proteins and a late age of onset [15]. Our study aimed to address the mechanisms linking protein toxicity to age, by inducing expression of a transgene encoding the toxic Arctic Aβ42 peptide at different ages and measuring the effects on levels of the toxic peptide and the extent of the resulting pathology.

Although the inducible GeneSwitch (GS) system has been widely used to study both ageing [26] and neurodegeneration [22], most studies have examined the effects of chronic induction. Characterisation of the dynamics of Aβ42 expression using our GS-inducible AD model [21] was, therefore, required. The responses of Aβ42 mRNA and peptide to cessation of induction differed, with a rapid return to baseline levels of mRNA and soluble Aβ42 expression, but insoluble Aβ42 peptide levels remained stable for several weeks. Importantly, switching-off transgene expression in young flies did not reverse Aβ42 toxicity, because both subsequent climbing ability and survival were reduced in a dose-dependent manner, probably attributable to the persistence of the highly stable, insoluble Aβ42 peptide. Studies using inducible APP mouse models of AD are congruent with these observations, because APP expression and Aβ production are suppressed upon removal of the inducer doxycyline, but the mice retain a considerable Aβ load for the following six months [27], [28]. The high stability of insoluble Aβ42 in our fly model may be related to the ability of the Arctic mutation to promote rapid aggregation [29]. The Z_Aβ3_ Affibody, a conformation-specific Aβ binding protein that prevents Aβ42 aggregation, clears Aβ42 and rescues toxicity when co-expressed with Arctic Aβ42 in *Drosophila* neurons [30], suggesting that aggregated Aβ42 can indeed induce neuropathological phenotypes in the fly.

We found that Aβ42 toxicity is related to the concentration of the peptide, with a dose-response in negative geotaxis and lifespan, in agreement with previous studies in cells showing that toxicity is dependent upon the dose of Aβ42 [31]. Importantly, when Aβ42 mRNA levels were equalised at different ages of induction, Aβ42 peptide accumulated to higher levels in older-induced flies, and subsequently shortened lifespan only when induced at later time-points. This difference correlated with an alteration in protein turnover with age [24]. Measurement of ^35^S-Methionine incorporation showed that general protein translation was reduced in older flies, consistent with published studies showing that the rate of protein synthesis decreases with age in a wide variety of cells, tissues, organs and organisms, including humans (reviewed in [25]). As there is no pronounced reduction in protein mass with age [32], this reduction in protein synthesis must be accompanied by a reduction in protein degradation, and indeed we found that proteasome activity declined with age. Reduced autophagy and activity of Aβ42-degrading enzymes such as insulin degrading enzyme [33], [34] and neprilysin [35] could also potentially contribute to a reduced rate of degradation of Aβ42 peptide at later ages.

We then examined the intrinsic effect of age on vulnerability to protein toxicity by inducing a fixed level of Aβ42 peptide in flies of different ages. Due to the persistence of Arctic Aβ42 peptide, at the end of the oldest induction period all treatment groups expressed the same level of Aβ42 at the same age. Measuring lifespan from this time-point only (44 days of age) revealed that those flies exposed to Aβ42 peptide for longer had a reduced survival, suggesting that the connection between age and Aβ42 toxicity, in part, reflects an accumulation of damage over time. However, in order to isolate the effect of age specifically on Aβ42 toxicity we controlled for the different durations of exposure by measuring survival from the time of transgene induction, and for the direct effect of ageing on lifespan by measuring the proportional reduction in survival compared to un-induced flies of the same age. This may explain why our conclusions differ from those of an earlier study, using a similar experimental design [36]. At standardised levels of mRNA expression, Ling et al., observed a shorter maximum lifespan in young-induced flies compared to old-induced flies, as did we, but concluded that younger flies were therefore more vulnerable to Aβ42 toxicity. However, the effects of age on protein turnover and survival were not considered, and our study highlights the importance of controlling for effects of age on protein turnover, duration of exposure and the direct effect of ageing on the output variable when investigating the relationship between ageing and protein toxicity. Our analysis showed that the relative survival of older flies was more reduced than that of young flies by an equivalent dose of Aβ42 peptide, indicating that ageing itself increased their vulnerability. Moreover this age-dependent increase in susceptibility appeared to be independent of the rate of Arc Aβ42 aggregation, suggesting that the relationship between age and the onset of proteotoxicity is not only due to the time required for an otherwise asymptomatic protein to become pathogenic.

Chronic over-expression of Arctic Aβ42 was also more toxic to older flies, despite a significantly lower duration of exposure, maximum concentration and lifetime Arctic Aβ42 burden. Thus, in all instances, either the equivalent or a lower Arctic Aβ42 load resulted in relatively higher levels of toxicity per time of exposure in older flies, demonstrating that ageing does increase vulnerability to protein-mediated toxicity. These results agree with other studies demonstrating that older neurons are more vulnerable to extracellularly applied Aβ42 fibrils both in cell culture [31] and in the brain of rhesus monkeys [37]. Our study builds upon these findings by providing the first evidence that this is the case *in vivo* under physiological conditions of Aβ42 expression and aggregation.

Our study suggests that, in addition to an accumulation of damage over time, ageing results in lowered degradation of a toxic protein and in increased vulnerability to its toxic effects. Hence, both the duration of exposure to toxic protein and the ageing process itself may contribute to making age the main risk factor for protein toxicity and neurodegeneration.

## Materials and Methods

### Fly stocks and husbandry

All fly stocks were maintained at 25°C on a 12∶12-h light:dark cycle at constant humidity on a standard sugar-yeast (SY) medium (15 gl^−1^ agar, 50 gl^−1^ sugar, 100 gl^−1^ autolysed yeast, 100 gl^−1^ nipagin and 3 ml l^−1^ propionic acid). Adult-onset neuronal-specific expression of Arctic mutant Aβ42 peptide was achieved by using the elav GeneSwitch (elavGS)-UAS system [GAL4-dependant upstream activator sequence; ElavGS was derived from the original elavGS 301.2 line and obtained as a generous gift from Dr H. Tricoire (CNRS, France). UAS-ArcAβ42 was obtained from Dr D. Crowther (University of Cambridge, UK)]. elavGS and UAS-lines were backcrossed six times into the w1118 genetic background. Male flies expressing UAS-constructs were crossed to female flies expressing elavGS, and adult-onset neuronal expression induced in female progeny by treatment with mifepristone (RU486; at indicated concentrations) added to the standard sucrose yeast (SY) medium.

### Lifespan analyses

Flies were raised at a standard density on SY medium in 200 mL bottles. Two days after eclosion once-mated females were transferred to experimental vials containing SY medium with or without RU486 (at indicated concentrations) at a density of 10 flies per vial. Deaths were scored and flies were transferred to fresh food 3 times a week. Data are presented as cumulative survival curves, and survival rates were compared using log-rank tests. To control for the effects of duration of exposure to Arc Aβ42 peptide and the direct effects of ageing on survival, survival from the age of induction was expressed as a percentage of non-RU-treated controls of the same age. Relative survival was compared between groups using the Wilcoxon matched pairs sign rank test.

### Negative Geotaxis Assays

To characterise effects of Arctic Aβ42 peptide on neuronal function, climbing assays were performed according to previously published methods [38]. Climbing ability was analysed every 2–3 days post-RU486 treatment. Briefly, 15 adult flies were placed in a vertical column (25 cm long, 1.5 cm diameter) with a conic bottom end, tapped to the bottom of the column, and their subsequent climb to the top of the column was observed. Flies reaching the top and flies remaining at the bottom of the column after a 45 sec period were counted separately, and three trials were performed at 1 min intervals for each experiment. Scores recorded were the mean number of flies at the top (n_top_), the mean number of flies at the bottom (n_bottom_) and the total number of flies assessed (n_tot_). A performance index (PI) defined as ½(n_tot_ + n_top_ – n_bottom)/_ n_tot_) was calculated.

### Quantification of total or soluble and insoluble Aβ42

Total Aβ42 was extracted based on previously published methods [30]. Five fly heads were homogenised in 50 µl GnHCl extraction buffer (5 M Guanidinium HCl, 50 mM Hepes pH 7.3, protease inhibitor cocktail (Sigma, P8340) and 5 mM EDTA), centrifuged at 21,000 g for 5 min at 4°C, and cleared supernatant retained as the total fly Aβ42 sample. Alternatively, soluble and insoluble pools of Aβ42 were extracted using a protocol adapted from previously published methods [39]: 50 fly heads were homogenised in 50 µl tissue homogenisation buffer (250 mM sucrose, 20 mM Tris base, 1 mM EDTA, 1 mM EGTA, protease inhibitor cocktail (Sigma)) then mixed further with 50 µl diethyl acetate (DEA) buffer (0.4% DEA, 100 mM NaCl and protease inhibitor cocktail). Samples were centrifuged at 135,000 g for one hour at 4°C (Beckman Optima^TM^ Max centrifuge, TLA120.1 rotor), and supernatant retained as the cytosolic, soluble Aβ42 fraction. Pellets were further resuspended in 200 µls ice-cold formic acid (FA; 70%), and sonicated four times for 15 sec on ice. Samples were re-centrifuged at 135,000 g for one hour at 4°C, then 100 µl of supernatant diluted with 1 ml FA neutralisation buffer (1 M Tris base, 0.5M Na_2_HPO_4_, 0.05% NaN_3_) and retained as the insoluble, formic acid-extractable Aβ42 fraction.

Total, soluble or insoluble Aβ42 content was measured in Arctic Aβ42 flies and controls using the High Sensitivity Human Amyloid Aβ42 ELISA kit (Millipore). Total Aβ42 samples were diluted 1∶100, and soluble versus insoluble Aβ42 samples diluted 1∶10 in sample/standard dilution buffer, then ELISA performed according to the manufacturers' instructions. Protein extracts were quantified using the Bradford protein assay (Bio-Rad laboratories Ltd, UK) and the amount of Aβ42 in each sample expressed as a ratio of the total protein content (pmoles/g total protein).

### Quantitative RT-PCR

Total RNA was extracted from 20–25 fly heads using Trizol (GIBCO) according to the manufacturers' instructions. The concentration of total RNA purified for each sample was measured using an *eppendorf biophotometer*. 1 µg of total RNA was then subjected to DNA digestion using DNAse I (Ambion), immediately followed by reverse transcription using the Superscript II system (Invitrogen) with oligo(dT) primers. Quantitative PCR was performed using the PRISM 7000 sequence-detection system (Applied Biosystems), SYBR Green (Molecular Probes), ROX Reference Dye (Invitrogen), and Hot Star Taq (Qiagen, Valencia, CA) by following manufacturers' instructions. Each sample was analysed in triplicate with both target gene (Arctic Aβ42) and reference gene (RP49, eIF-1A and αTubulin84) primers in parallel. The primers for the Aβ transgenes were directed to the 5′ end and 3′ end of the Aβ coding sequence: forward GATCCTTCTCCTGCTAACC, reverse CACCATCAAGCCAATAATCG. The reference gene primers were as follows: RP49 forward ATGACCATCCGCCCAGCATCAGG and reverse ATCTCGCCGCAGTAAACG; eIF-1A forward ATCAGCTCCGAGGATGACGC and reverse GCCGAGACAGACGTTCCAGA; αTubulin84 forward TGGGCCCGTCTGGACCACAA and reverse TCGCCGTCACCGGAGTCCAT [40].

### Proteasome activity

Fly heads were homogenized in 25 mM Tris, pH 7.5 and protein content determined by Bradford assay. Chymotrypsin-like peptidase activity of the proteasome was assayed using the fluorogenic peptide substrate Succinyl-Leu-Leu-Val-Tyr-amidomethylcoumarin (LLVY-AMC), based on a previously published protocol [41]. 20 µg of crude fly head homogenate total protein was incubated at 37°C with 25 µM LLVY-AMC in a final volume of 200 µLs. Enzymatic kinetics were conducted in a temperature-controlled microplate fluorimeter (Tecan Infinite M200), at excitation/emission wavelengths of 360/460 nm, measuring fluorescence every two minutes for 30 min. Proteasome activity was determined as the slope of AMC accumulation over time per mg of total protein (pmoles/min/mg).

### ^35^S-methionine incorporation

Protein translation was measured in fly heads using a method adapted from [42]. Standard SY medium was first supplemented with 100μCi ^35^S methionine/ml of food (American Radiolabeled Chemicals 1 mCi/37MBq ARS0104A). 15 flies were transferred to each vial containing 1 ml radioactive SY medium. After three-hours of feeding, flies were transferred to non-radioactive SY for 30 min in order to purge any undigested radioactive food from the intestines. Flies that were in contact with the radioactive food for 1 minute were used as a background control. Flies were then decapitated using liquid nitrogen and the heads homogenized in 1% SDS and heated for 5 minutes at 95°C. Samples were then centrifuged twice for 5 minutes at 16,000 g. Proteins were precipitated by the addition of the same volume of 20% cold TCA (10% TCA final concentration) and incubated for 15 minutes on ice. Samples were then centrifuged at 16,000 g for 15 minutes, the pellet washed twice with acetone and then resuspended in 200 μl of 4 M guanidine-HCl.

Samples (100 μls) were mixed with 3 mls of scintillant (Fluoran-Safe 2, BDH) and radioactivity counted in a liquid scintillation analyzer (TriCarb 2800TR, Perkin Elmer), with appropriate quench corrections. SDS-homogenates, prior to TCA precipitation, were also sampled and analysed as a measure of the total radioactivity (incorporated and un-incorporated) present. Total protein for each sample was determined by Bradford assay and a translation index was calculated as follows: (TCA protein cpm/total cpm)/µg protein per sample.

### Statistical analyses

Data are presented as means ± SEM obtained in at least three independent experiments. Log-rank, Wilcoxon matched pairs sign rank, analysis of variances (ANOVA) and Tukey's HSD (honestly significant difference) post-hoc analyses were performed using JMP (version 7.0) software (SAS Institute, Cary, NC, USA).

## Supporting Information

Figure S1 **Effects of RU486 treatment on lifespan of control flies.** Survival curves of elavGS/+, UAS-ArcticAβ42/+ and UAS-ArcticAβ42/+; elavGS/+ flies treated chronically with 200 µM RU486 from 2 days post-eclosion. Median lifespans (Log-rank test *P* values): elavGS/+, 73.5 days for −RU and 76 days for +RU 200 (*P* = 0.1306); UAS-ArcticAβ42/+, 71 days for −RU and 73.5 days for +RU 200 (P = 0.08); UAS-ArcticAβ42/+; elavGS/+ 71 days for −RU and 38.5 days for +RU 200 (*P*<0.0001).
(EPS)

Figure S2 **Arctic Aβ42 mRNA equalisation in young and old flies.** (A) Arctic Aβ42 mRNA expression levels in the heads of UAS-Arctic Aβ42/+; elavGS/+ females treated with 0, 50, 100 and 200 µM RU486, for 1 week, from 5 days and 20 days post-eclosion. Data are means ± SEM (n = 5 for each group) and were compared using two-way ANOVA and Tukey's HSD post-hoc analyses. There was no significant difference between young-induced flies at 50 µM and old-induced flies at 100 and 200 µM RU486 (*P*<0.05 comparing young-induced flies at 100 and 200 µM to all other conditions).
(EPS)

Figure S3 **Proteasome activity declines with age in control flies.** Proteasome activity in the heads of elavGS/+ flies treated with or without RU486 under the same conditions as described in Figure 2E. Data are presented as mean activities (pmoles/min/mg protein) ± SEM (n = 5). *** *P*<0.0002 comparing 9 versus 27 day-old flies, *P* = 0.635 comparing + versus – RU486 (two-way ANOVA); *P*<0.05 comparing RU 50 [2–9d] to RU 200 [20–27d] flies and –RU day 9 to –RU day 27 flies (Tukey's HSD).
(EPS)

Figure S4 **Arctic Aβ42 protein equalisation in young and old flies.** Total (soluble and insoluble) Aβ42 protein levels in the heads of UAS-Arctic Aβ42/+; elavGS/+ females pulsed with various RU486 concentrations (50 µM–200 µM), for 1 or 2 weeks, from days 5, 20 and 30 post-eclosion. Arctic Aβ42 protein levels were quantified at the end of each pulse. Data are means ± SEM (n = 5 for each group) and were compared using two-way ANOVA and Tukey's HSD analyses for each induction period. There were no significant differences between 5 day and 20 day-old flies treated with 200 µM RU for 1 week, or 5 day old flies on 100 µM RU and either 20 or 30 day old flies on 200 µM RU for 2 weeks.
(EPS)

Figure S5 **Survival analysis of inducible Arctic Aβ42 flies treated either conditionally or chronically with RU486.** (A) Original survival curves of flies depicted in Figure 3B. UAS-Arctic Aβ42/+; elavGS/+ females were conditionally treated with 100 µM RU486 (RU) from 5-days and 200 µM RU486 from 20- and 30-days post-eclosion for 2 weeks only, and were maintained on −RU prior to and following the RU pulse. Arrows indicate the induction period, and Aβ42 protein levels were equalised at the end of the RU486 pulse (Fig 3A). Median lifespans were: 78 days for −RU, 57 days for +RU 100 [5–19d], 60 days for + RU 200 [20–34d] and 67 days for + RU 200 [30–44d]. Log rank test and *P* values: *P*<0.0001 comparing −RU versus +RU 100 [5–19d], + RU 200 [20–34d] and + RU 200 [30–44d], *P*<0.05 comparing +RU 100 [5–19d] versus + RU 200 [20–34d], and *P*<0.0001 comparing +RU 100 [5–19d] and +RU 200 [20–34d] versus + RU 200 [30–44d]. (B) Survival curves of day 44 survivors only from the experiment depicted in (A). Median lifespans were: 36 days for −RU, 18 days for +RU 100 [5–19d], 18 days for + RU 200 [20–34d] and 25 days for + RU 200 [30–44d]. Log rank test and *P* values: *P*<0.0001 comparing −RU versus +RU 100 [5–19d], + RU 200 [20–34d] and + RU 200 [30–44d], *P* = 0.08 comparing +RU 100 [5–19d] versus + RU 200 [20–34d] and *P*<0.0001 comparing +RU 100 [5–19d] and + RU 200 [20–34d] versus + RU 200 [30–44d]. (C) Relative survival of +RU 100 [5–19d] flies, expressed as a percentage of −RU controls and plotted from progressively later ages (5, 20 and 30d). Older flies exhibit a significantly greater reduction in relative survival in response to Arctic Aβ42 expression than younger flies (*P*<0.0001 comparing 20d and 30d with 5d survivors, and 20d with 30d survivors, Wilcoxon matched pairs sign rank test). (D) Original survival curves of UAS-Arctic Aβ42/+; elavGS/+ flies depicted in Figure 4C. Arrows indicate the age of induction, from which flies were treated chronically with 200 µM RU486. Median lifespans were: 75 days for −RU, 41 days for +RU 200 2d and 56 days for +RU 200 20d. Log rank test and *P* values: *P*<0.0001 comparing −RU versus +RU 200 2d and +RU 200 20d, and +RU 200 2d versus +RU 200 20d.
(EPS)

Table S1 **Details of RU486 pulse conditions used in acute induction experiments.** Note that these are days post-eclosion and that all flies were treated with RU486 at the same age (day 2 post-eclosion).
(DOC)
