# Supplementary material for: Ageing Increases Vulnerability to Aβ42 Toxicity in Drosophila
Source: PLoS One. 2012 Jul 12;7(7):e40569. doi: 10.1371/journal.pone.0040569 (PMC3395685; doi:10.1371/journal.pone.0040569)
Supplement: Table S1 — Details of RU486 pulse conditions used in acute induction experiments. Note that these are days post-eclosion and that all flies were treated with RU486 at the same age (day 2 post-eclosion). (DOC) [file pone.0040569.s006.doc]

| **Treatment** | **Day switched to + RU** | **Day switched to -RU** |
| --- | --- | --- |
| -RU | n/a | n/a |
| +2d RU | 2 | 4 |
| +4d RU | 2 | 6 |
| +7d RU | 2 | 9 |
| + 14d RU | 2 | 16 |
| +RU (chronic) | 2 | n/a |
